# Consequences of school closures due to COVID-19 in DRC, Nigeria, Senegal, and Uganda

**DOI:** 10.1371/journal.pgph.0002452

**Published:** 2023-10-16

**Authors:** Rawlance Ndejjo, Andrew K. Tusubiira, Suzanne N. Kiwanuka, Marc Bosonkie, Eniola A. Bamgboye, Issakha Diallo, Steven N. Kabwama, Landry Egbende, Rotimi F. Afolabi, Mamadou Makhtar Mbacké Leye, Noel Namuhani, Yves Kashiya, Segun Bello, Ziyada Babirye, Ayo Stephen Adebowale, Marieme Sougou, Fred Monje, Susan Kizito, Magbagbeola David Dairo, Omar Bassoum, Alice Namale, Ibrahima Seck, Olufunmilayo I. Fawole, Mala Ali Mapatano, Rhoda K. Wanyenze

**Affiliations:** 1 Department of Disease Control and Environmental Health, School of Public Health, College of Health Sciences, Makerere University, Kampala, Uganda; 2 Department of Community Health and Behavioural Sciences, Makerere University School of Public Health, Kampala, Uganda; 3 Department of Health Policy, Planning and Management, School of Public Health, College of Health Sciences, Makerere University, Kampala, Uganda; 4 Department of Nutrition, Kinshasa School of Public Health, Kinshasa, Democratic Republic of Congo; 5 Department of Epidemiology and Medical Statistics, Faculty of Public Health, College of Medicine, University of Ibadan, Ibadan, Nigeria; 6 Department of Public Health, Faculty of Health Sciences, University Amadou Hampaté Ba, Dakar, Senegal; 7 Department of Preventive Medicine and Public Health, Faculty of Medicine, Pharmacy and Dentistry, University Cheikh Anta Diop of Dakar, Dakar, Senegal; 8 Department of Biostatistics and Epidemiology, Kinshasa School of Public Health, Kinshasa, Democratic Republic of Congo; ICMR - National Institute of Epidemiology, Chennai, India

## Abstract

In 2020 and 2021, Governments across the globe instituted school closures to reduce social interaction and interrupt COVID-19 transmission. We examined the consequences of school closures due to COVID-19 across four sub–Saharan African countries: the Democratic Republic of Congo (DRC), Nigeria, Senegal, and Uganda. We conducted a qualitative study among key informants including policymakers, school heads, students, parents, civil society representatives, and local leaders. The assessment of the consequences of school closures was informed by the Diffusion of Innovations theory which informed the interview guide and analysis. Interview transcripts were thematically analysed. Across the four countries, schools were totally closed for 120 weeks and partially closed for 48 weeks. School closures led to: i) Desirable and anticipated consequences: enhanced adoption of online platforms and mass media for learning and increased involvement of parents in their children’s education. ii) Desirable and unanticipated consequences: improvement in information, communication, and technology (ICT) infrastructure in schools, development and improvement of computer skills, and created an opportunity to take leave from hectic schedules. iii) Undesirable anticipated consequences: inadequate education continuity among students, an adjustment in academic schedules and programmes, and disrupted student progress and grades. iv) Undesirable unanticipated: increase in sexual violence including engaging in transactional sex, a rise in teenage pregnancy, and school dropouts, demotivation of teachers due to reduced incomes, and reduced school revenues. v) Neutral consequences: engagement in revenue-generating activities, increased access to phones and computers among learners, and promoted less structured learning. The consequences of school closures for COVID-19 control were largely negative with the potential for both short-term and far-reaching longer-term consequences. In future pandemics, careful consideration of the type and duration of education closure measures and examination of their potential consequences in the short and long term is important before deploying them.

## Introduction

The Coronavirus disease 2019 (COVID-19) pandemic presented a global challenge of developing appropriate interventions to quickly respond to the fast-spreading novel virus [1, 2]. Initial global recommendations for COVID-19 control were based on restrictions on travel and mass gatherings [3, 4]. Consequently, some governments implemented national-wide school closures to reduce social interaction and interrupt COVID-19 transmission [5]. At the peak of the pandemic in 2020, about 1.6 billion students in 180 countries had been affected by school closures worldwide [6].

In Africa, school closures were implemented as early as February 2020 affecting more than 250 million students, adding to about 100 million out-of-school children before the pandemic [7]. Apart from Burundi, all countries in sub-Saharan Africa (SSA) closed their schools with only about a third of the countries partially reopening them within a hundred days of initial closure [8]. With successive COVID-19 waves across the continent, school closures lasted more than 20 weeks with periodic and stringent measures in several countries. By 2021, Uganda recorded the longest duration of school closures of 83 weeks worldwide [6].

The closure of schools had several effects, some of which had not been foreseen. The unprecedented and abrupt interruption of learning risked reversing earlier gains made in increasing school attendance and quality of education in Africa [9]. Recent evidence predicted that short-term COVID-19-related learning losses were up to one year in sub-Saharan Africa and that the learning loss could accumulate to 2.8 years [10, 11]. Beyond the academic losses, school closures led to a setback in students’ emotional well-being due to limited social interaction; increase exposure and vulnerability to domestic abuse; irregular or unhealthy diets; unintended pregnancies and high rates of socioeconomic and gender disparities [12, 13]. Moreover, school closures could have had broader, far-reaching short- and long-term unexplored effects on parents, teachers, communities, and schools among others. Efforts by most governments were targeted at dealing with the anticipated disruptions in learning and scanty information exists to holistically document the consequences of school closure in SSA. We examined and documented the consequences of school closures due to COVID-19 in the Democratic Republic of Congo (DRC), Nigeria, Senegal, and Uganda to provide a broader understanding of the consequences of school closures and the mitigation measures implemented. Findings should inform considerations for deployment of school closures in epidemic control.

We used Roger’s Diffusion of Innovations theory [14] to explore, organize and document the consequences of school closures. The theory describes the process of adoption of innovations within a social system and provides a taxonomy for categorizing resultant consequences [14]. We explored the consequences of school closures as the new intervention that had been introduced for COVID-19 control. The theory proposes four factors that influence the consequences due to innovation introduction and these are: (i) nature of the mitigation measure (school closures), (ii) use of the mitigation measure, (iii) characteristic of members and (iv) nature of the social system. Consequences can be intended or unintended and are classified based on whether they are desirable or undesirable, direct, or indirect and anticipated (could have been predicted beforehand, had adequate efforts been made) or unanticipated. Consequences that are desirable and anticipated are mostly intended and the rest are unintended [15–18]. Guided by the twelve steps in evaluating unintended consequences [19], we considered the decision makers perspective to determine what was anticipated or “foreseeable”.

## Methods

### Study setting and design

This study was conducted in four countries in SSA: DRC in Central Africa; Nigeria and Senegal in West Africa; and Uganda in East Africa. Of Nigeria’s 206 million people [20], at least 32.8% are school-going children. Senegal has a population of 16.7 million of whom 40.7% are school-going [21]. In DRC, 43.4% of the country’s 83 million people are school-going children [22]. In Uganda, the school-going population constitutes 49.4% of the country’s 41.6 million people [23]. This study was qualitative and involved key informant interviews among stakeholders who shared their perspectives and experiences on the consequences of school closures and measures undertaken to mitigate them.

### Interviews

We conducted interviews among key informants (KIs) using a pretested interview guide (SI Interview Guide) informed by the Diffusion of Innovations theory. The guide had several questions that explored the nature of school closures and their implementation; those impacted including their norms, culture, and characteristics; the effects resulting from the closure to the school, community, and population; and strategies employed to minimize the negative effects of the closure. The KIs were purposively selected and included policymakers from the Ministry of Education, Ministry of Health, school heads, students, parents, civil society representatives, and local leaders. The KIs shared their perspectives regarding the consequences of school closures in their communities, districts, or nationally with consideration of differences by education levels, region, gender, and urban and rural areas. The KI interviews were conducted by members of the research team either physically (DRC) or virtually over Zoom or through phone calls due to the COVID-19 restrictions. Interviews were conducted in English (for Nigeria and Uganda) and French (for DRC and Senegal) by at least two members of the research team who were graduates of public health and had experience in qualitative interviewing. One research team member asked questions while the other took notes. Interviews lasted about an hour. The number of interviews conducted in each country followed the principle of saturation. The KI interview guide was originally designed in English and a final version was translated into French. All contacted KIs agreed and participated in the study.

### Data management and analysis

All KIIs were audio-recorded and transcribed verbatim in English or French. The KII transcripts were synthesized and analysed separately for each country, and results were organized around themes following the Diffusion of Innovations theory. The analysis of transcripts involved generation of a codebook for each country based on which data were coded and thematically analysed and presented per the guiding framework. Following analysis, all countries wrote their results in English, and these were then synthesized across the four countries. The presentation of results is supported by quotations from key informant interviews and was guided by the Consolidated criteria for reporting qualitative research guidelines.

### Ethical considerations

This study received ethical approval from Research and Ethics Committees in the different countries. In DRC, approval was granted by the Kinshasa School of Public Health Ethics Committee (ESP/CE/198/2020). In Nigeria approval was granted by the National Health Research Ethics Committee while in Senegal, the National Committee of Ethics and Research provided ethics permission. In Uganda, the study was approved by the Makerere University School of Public Health Higher Degrees, Research and Ethics Committee (HDREC #903)) and registered by the Uganda National Council for Science and Technology (UNCST #HS1121ES). Written informed consent was sought in DRC where physical interviews were conducted while verbal consent was obtained in the other countries before interviewing informants. All study data were confidentially managed.

## Results

### Overview of school closures due to COVID-19

In all four countries, school closures were implemented to minimize COVID-19 spread. In DRC, the first school closure was announced on March 19^th^, 2020, initially intended for four weeks but went on for six months till September 2020. During the COVID-19 second wave, another school closure was announced on December 18^th^, 2020, and classes resumed on February 22^nd^, 2021. In Nigeria, schools were first closed on March 23^rd^ 2020 and reopened in June 2020 for those writing their examinations. Thereafter, there was a partial school reopening from September 21^st^ to November 1^st^, 2020, and a full reopening henceforth. Senegal’s school closure started on March 14^th^, 2020, for three months. In Uganda, the first closure happened on March 20^th^, 2020, with partial re-opening in October 2020. Schools were then closed again in June 2021 until January 2022 when a full reopening was done. Overall, Uganda had the longest school closure with 66 weeks of full and 23 of partial closure while Senegal had the least including 12 weeks of full and 10 weeks of partial closure as of March 31^st^ 2022 [24] (Table 1).

**Table 1:** Overview of school closures across the four countries.

Country	Date of 1^st^ confirmed COVID-19 case	Period of 1^st^ full closure	Period of 2^nd^ full closure	Enrolment^#^	School-age population^#^	Weeks fully closed^#^	Weeks partially open^#^
DRC	March 10^th^, 2020	March 19^th^ to September 2020	December 18^th^ to February 22^nd^ 2021	21,899,644	36,049,677	24	9
Nigeria	February 27, 2020	March 23^rd^ to September 20^th^ 2020	Not applicable	37,926,644	67,595,980	18	6
Senegal	March 2, 2020	March 16^th^ to July 1^st^ 2020	Not applicable	3,574,141	6,804,878	12	10
Uganda	March 22^nd^ 2020	March 20^th^ to October 2020	June 2021 to January 2022	10,247,892	20,573,918	66	23

^#^ UNESCO Institute of Statistics Global Monitoring of School Closures Caused by the COVID-19 Pandemic, available at: https://covid19.uis.unesco.org/global-monitoring-school-closures-covid19/

### Consequences of school closures

We conducted 102 KI interviews across the four countries as follows: DRC (22), Nigeria (24), Senegal (20) and Uganda (36). The consequences of school closures have been organized across five themes. These are: i) desirable and anticipated consequences, which constitute intended consequences, and ii) desirable and unanticipated, iii) undesirable anticipated and iv) undesirable unanticipated which are the unintended consequences. The others are neutral consequences. These consequences are summarized in Table 2.

**Table 2:** Consequences of school closures in DRC, Nigeria, Senegal, and Uganda.

Type of consequence	Anticipated (Changes are recognized by the members of a social system / could have been predicted)		Unanticipated (Changes are not recognized by the members of a social system / could not have been predicted)	
	Direct (Changes as an immediate response to school closures)	Indirect (Changes as second order result of the effects of school closures)	Direct (Changes as an immediate response to school closures)	Indirect (Changes as second order result of the effects of school closures)
**Desirable** (Effects of the measure tend to be functional for the social system (i.e., effects producing additional benefits, helping the system work properly)	• Enhanced adoption of virtual teaching and learning. • Use of mass media for learning and improved access to materials.	• Increased involvement of parents in their children’s education.	• Improvement in information, communication, and technology infrastructure in schools. • Development and improvement of computer skills.	• Opportunity to take a break from busy schedules. • Increased funding for hygiene improvement in schools. • Improved hygiene and reduced illness among children. • Increased earning among some teachers from businesses Improved Technology / ICT skills through E-learning. • Improved family bondage and parental guidance. • Opportunity to listen and watch lesson recordings repeatedly for better comprehension. • Increased local and international collaboration among teachers.
**Neutral** (effect is neither positive or negative)	• Schedules for online classes and teaching were less structured.	• Increase in home schooling and coaching by teachers.	• Increased access to phones and computers among children.	• Engagement in revenue-generating activities among children. Increased screen time among children.
**Undesirable** (effects of a mitigation measure tend to be dysfunctional (i.e., negative, causing harm, not helping the system work properly).	• Prolonged lack of studying for many students. • Reduced opportunities for development and improvement. • Loss of personal contact for the physically challenged that make use of braille in learning. • Academic performance of students deteriorated. • Decline in the discipline of students.	• Socioeconomic hardship on the school owners and teachers because of discontinuity of salaries. • Students missed out on social interactions with their mates.	• Private schools’ lost revenue due to less enrolment and school dropouts. • Reduced number of students affected incomes at schools. • Aggravated lack of income among private school teachers. • Disruption of school curriculum and slowed learning. • Poor concentration among students for online classes, • Struggle to learn and keep up with technology among pupils, teachers, and parents. • Loss of interest in education among students as some students became business oriented. • Reduced employment in schools (Some lost jobs, other driven into dual practice). • Stalling of institutional projects and unexpected expenses for school maintenance. • Vandalism of school properties. • Increased alcohol and substance use among students. • Public and private inequalities in provision of resources for virtual classes. • For Children with disabilities, parents could care for them and slowed cognitive development.	• Teachers lost interest/ motivation in their profession and work. • Decreased motivation of other school staff. • Poor living standards and depression among teachers due to loss of incomes. • Domestic violence and marriage break-ups in homes of teachers due to financial constraints. • Increased teacher-workload after lockdown. • High pregnancy and sexual violence. • Engagement in transactional sex and increase in sexual violence. • Reduced access to basic needs including food. • High school dropouts. • Sale of private schools or loss. school assets to meet bank loans. • Massive shutting down of private schools. • Conversion of school premises to alternative use • Difficulties paying tuition among parents. • Difficulty juggling home and work responsibilities.

** The unintended consequences are shaded

### Desirable and anticipated consequences

School closures led to enhanced adoption of online platforms and mass media for learning and increased involvement of parents in their children’s education.

#### Enhanced adoption of virtual teaching and learning

Across all countries, school closures led to enhanced adoption of e-learning and the use of mass media to ensure continuity of learning. This cut across all levels of education from primary to tertiary, for example, in Nigeria, a Schoolgate e-Learning service and mobile classroom was created for primary school pupils offering curriculum-based education. In most countries, e-learning was adopted wholly or partially at the tertiary level as the mode of teaching. However, with poor internet infrastructure, e-learning was mostly accessible to those in urban areas with better connectivity and more reliable electricity access. The intervention also mostly favoured those of middle to high socioeconomic status who could afford it.

#### Use of mass media for learning and improved access to materials

To bridge gaps in access to the internet and e-learning, especially among rural underserved communities, the governments used mass media to reach learners and/or distributed learning materials. Some organizations also organized communal learning sessions and distributed radios to support learning. In DRC, the education minister organized television courses in some underserved areas including Kasai province but this had very limited effect in some areas such as Kananga city with sparse electricity coverage leading to students being unable to follow courses. The distribution of learning materials including pamphlets and workbooks bridged such gaps. However, materials for children with special needs such as braille for the blind were not distributed.

#### Increased involvement of parents in their children’s education

Among the indirect consequences, there was increased involvement of parents in the education of their children including supporting their learning and progression which improved family bonding. Parents also spent a significant amount of time with their children engaging in extracurricular activities beyond schoolwork. These activities included cooking, cleaning, gardening, and playing games building their practical knowledge and skills.

### Desirable and unanticipated consequences

School closures contributed to improvement in information, communication, and technology (ICT) infrastructure in schools, development and improvement of computer skills, and created an opportunity to take leave from hectic schedules. There was also improved hygiene and reduced illnesses among children.

#### Improvement in information, communication, and technology infrastructure in schools

Due to the need to ensure continuity of learning mostly through e-learning, several schools across the different countries upgraded their information, communication, and technology (ICT) infrastructure including computers, phones, and internet access. This, however, only happened in a few wealthy urban schools. On the other hand, some companies donated ICT equipment to schools to support e-learning though this was on a small scale.

#### Development and improvement of computer skills

School teachers, parents and pupils further developed and improved their computer skills especially in using computer tools and applications such as Zoom, Microsoft Teams and Google Classroom. Students also adopted tools like WhatsApp to connect with peers and their teachers improving information exchange.

“*There was adoption of new technology*. *Before the lockdown*, *I knew about Zoom but I wasn’t well conversant with it*. *I had to learn how to use Zoom and Google Classroom*. *The pandemic was a rejuvenation of social media and discussions among people via the internet and other platforms*.*”* (Postgraduate student, Nigeria)

#### Opportunity to take a break from busy schedules

Some teachers, especially at tertiary institutions and school superintendents said that the COVID-19 containment measures such as lockdowns and school closures were an opportunity to obtain leave because they usually worked throughout the year. They mentioned that the break also provided them with some time to focus on their research without being interrupted by students and other academic tasks.

*"The confinement allowed us to be more focused on personal research*, *as no students were interrupting us*. *Additionally*, *it had been a long time since we had a holiday at the university with overtime and overlapping academic years*. *This confinement allowed us to rest for a while and do research*.*"* (University lecturer, DRC).

#### Improved hygiene and reduced illness among children

A positive but indirect consequence following the closure and reopening of schools was an improvement in hygiene and sanitation at schools and in the community. Parents also reported a reduction in the occurrence of illnesses, especially those of a respiratory nature among children during their stay at home.

“*Among the positive effects*, *it has improved hygiene in schools as the government gave us materials such as hand washing facilities which we will continue to use to maintain hygiene*.” (Primary teacher, Uganda).

### Undesirable and anticipated consequences

The closure of schools led to inadequate education continuity among students, an adjustment in academic schedules and programmes, and derailed student progress and deteriorated their grades.

#### Inadequate education continuity among students

Even though there was continuity of learning in some schools and among some students, overall, a large proportion of learners did not engage in any learning activities. Indeed, inequalities in the provision of resources for virtual classes existed between private and urban schools compared to public and rural ones, widening learning outcomes between students. Students with disabilities were also negatively impacted, for example, blind students who make use of braille in learning did not receive specialized materials and could not join the online/virtual classes.

*Online learning only helped parents who are in urban places*, *but not those in rural areas with children who do not even know what a smartphone is*. *Even with the use of television*, *many in rural areas did not have access to one to keep up with their urban counterparts*. *I think they remained back in their learning”* (Parent, Uganda).

#### Adjustment of academic schedules and programmes

School closures and uncertainties caused by COVID-19 led the government and most institutions to adjust academic calendars and programmes which negatively impacted progress for learners, teachers’ ability to effectively plan their classes, and broader school plans. Parents and students were also uncertain about the school calendar, making it hard to plan appropriately. Changes in school teaching approaches including online learning also led to disruptions in learning processes among learners and compromised teaching quality. On return to school, several students experienced an overload of courses and shortened study periods to catch up with uncompleted courses following school closures. The teachers also had bigger classes with several students who had remained at the preceding levels being joined by others.

*"I have a lot of lessons to learn when in class*. *My classmates are also no longer concentrating during online sessions as they would in physical classes*.*”* (Student, Senegal)

#### Lack of academic progression and deterioration of student grades

There was slowed study progress for students due to delays in getting learning materials, poor concentration with online classes, and reduced learning supervision. Consequently, students’ grades and discipline deteriorated. Some students lost interest in education and their academic performance deteriorated while others became business oriented. Some parents attributed the decline in academic performance to the prolonged stay at home without studying and the distractions children faced including playing and watching television. According to some teachers interviewed, the observed drop in grades was linked to an insufficient hourly volume of studying. There was also a struggle among learners, teachers, and parents to learn and keep up with technology.

*"The academic performance of children has been falling for years and*, *with the situation has become worse due to COVID-19*. *This deplorable situation has further lowered the grades of children*.*"* (Secondary school teacher, Senegal)

### Undesirable and unanticipated consequences

Among teachers, especially in private schools, closures led to their demotivation due to reduced incomes. Private schools in particular also experienced a reduction in their revenue and some became bankrupt and were sold or repurposed for other businesses, further compromising students continuation of learning. Among learners, there was a reported increase in sexual violence including engaging in transactional sex, a rise in teenage pregnancy, and school dropouts.

#### Demotivation and loss of teachers

School closures led to demotivation and reduced incomes among teachers, especially in private schools due to lack of pay for an extended period. Some teachers were forced to shift momentarily to informal businesses including engaging in menial and manual jobs to supplement their income. KIs noted that some teachers lost jobs which led to poorer living standards. The poor financial situation among teachers contributed to instances of domestic violence and marriage break-ups. Even when schools resumed, several teachers who had left the profession opted to concentrate on their new income-generating activities than returning to class which led to the loss of experienced teachers. Those who remained in the schools also complained of an increased workload due to the departure of their colleagues. The demotivation cut across other school staff including the non-teaching staff.

“W*e witnessed a number of them [teachers] losing lives because of school closures*. *Some committed suicide because they were unable to take care of their family needs and all that…*. *it has not been an easy time for the teachers…* … *it caused a lot of domestic violence generally because normally when there is no income*, *and you are all home*, *such issues arise*.” (Teacher, Uganda).

#### Reduced revenue, bankruptcy, and sale of schools

School closures led to a loss of revenue among schools as they did not generate revenue for a long period. Several private schools had accumulated loans that they were unable to service leading to some being sold off or repurposed to generate income including as rental houses, lodges, or other businesses. Even when schools were reopened, several repurposed buildings were not reverted to serve as schools. Following school closures, enrolment was low in some areas which coupled with school dropouts led to reduced revenue.

#### Engagement in transactional sex and increase in sexual violence

The pandemic caused an economic crisis which increased the exposure of girls to transactional sex including with multiple partners to meet their needs. KIs said that school closures led to an increase in sexual activity among adolescents including transactional sex with concurrent partners which increased the risk of unwanted pregnancies, sexually transmitted infections, and practices of clandestine abortions. KIs also reported an increase in sexual violence especially rape.

“*I think that even the pregnancy rate*, *uh* … *if we could make statistics today*, *we would see that the rate of early pregnancy is quite high*, *why*? *Because during that time*, *young people didn’t go out to schools*, *churches*, *or markets*. *With the sexual instinct that is at its peak*, *especially among adolescents and young people so uh* …*that’s how* … *there are probably early pregnancies*, *sexually transmitted infections*, *and even HIV*, *and that’s another problem*.” (Programme Manager, Ministry of Health, DRC)

#### Rise in teenage pregnancy

In all four countries, a rise in teenage pregnancies was reported which contributed to the high school dropouts registered. School closures removed the structured environment and safety net that generally ensured the protection of girls. Unlike the school system where students are under supervision for the great part of the year, parents were unable to regularly supervise their children and had few opportunities to provide sexual health education. This led to increased risk of teenage pregnancy and child marriage.

#### Dropouts of learners

School closures led to dropouts in all study countries and girls were disproportionately affected due to pregnancy and early marriages. Other children, especially boys engaged in petty trade distracting them from school, especially in low-income households.

#### Increased alcohol consumption, drug use, and social vices

KIs also noted that there was increased use of alcohol and drugs, especially among teenagers who stayed at home due to heightened access, boredom, and peer influence. Families with teenagers faced a more difficult situation finding extra activities to keep their children busy and provide for the above-average consumption of food and use of household items. Thus, street begging, abuse of drugs and homelessness, and an increase in youth delinquency were reported, hugely affecting the family unit.

*“If there are no online courses*, *you stay at home and you take part in hobbies*, *card games*. *Some teenagers indulged in alcohol abuse and urban banditry*.*”* (Student, DRC).

#### Reduced socialization among children

Measures to control COVID-19 and keep children out of school also impacted socialization among children who had been used to a culture of sharing and working together and were now supposed to be on their own. Some learners experienced loneliness and boredom as they could not see their friends.

*Nigeria is a communal society where we greet and hug each other so that had to be excluded*. *For children who had to play*, *we had to pack up toys and started telling them not to share their things*. *It was very difficult for them to have that understanding but due to the awareness*, *they understood the situation*.*”* (School Administrator, Nigeria)

#### Difficulty juggling home and work responsibilities

As the pandemic came along with the need for parents to stay home with their children, most found it hard to look after their children and go to work, especially also with closed daycare centres. Moreover, parents also had to invest more time to support their children’s learning, reducing their work time and with children around, those who worked from home found it hard to concentrate and be productive.

### Neutral consequences

The neutral consequences of school closures recorded were engagement in revenue-generating activities, increased access to phones and computers among learners, and reduced structure to learning and increase in homeschooling.

#### Engagement in revenue-generating activities among learners

Due to school closures, students engaged in other revenue-generating activities during the lockdown including selling masks and airtime sometimes to support their families to make ends meet. KIs noted that some students preferred continuing to work and earning money for themselves and their families over returning to school.

#### Increased access to phones and computers among learners

The move toward e-learning led to several parents buying phones and computers to support their children’s learning. The increased access to mobile phones also led to an increase in the use of social media tools such as WhatsApp which supported organizing students and learning. Overall, there was also increased screen time among students bordering on cyber addiction.

#### Less structured learning and increase in homeschooling

As learners were mostly at home, they had more flexible schedules for their online classes or extracurricular activities. There was also an increase in homeschooling of students and coaching of learners by teachers and other tutors, which were usually less structured.

## Discussion

This study documented the consequences of school closures due to COVID-19 in DRC, Nigeria, Senegal, and Uganda through key informant interviews with various stakeholders. Overall, the negative consequences of school closures outweighed the positive effects across the four countries. While school closures led to the adoption of innovative methods of learning (online and distance learning), parental involvement in children’s education and increased engagement in students’ extracurricular activities, it also impacted learner’s progress, altered academic schedules, affected children’s discipline, and contributed to school dropouts. School closures also negatively impacted teacher welfare with some leaving the profession and it led to bankruptcy of schools due to reduced revenue.

The closure of schools led to drastic changes in the mode of teaching from physical attendance to online or mass media classes leading to improved knowledge and skills in information technology among both learners and teachers. Learning was localized at home and parents were involved in their children’s learning and teaching efforts more than before. Although by 2015, online education was growing in sub-Saharan Africa, mainly in universities [25], school closures contributed to its greater popularity among educational institutions at all levels in the region [26]. However, there were gaps in access to elearning between rural and urban areas and low- and middle-income households which further contributed to learning disparities. Online training was also characterized by difficulties for students to comprehend concepts being taught, less attention during the teaching, inconsistent access to internet connectivity, lack of resources, and eventually lower students’ performance [27, 28] and satisfaction [29]. The use of mass media and distribution of learning materials supported bridging access gaps, but other learners were still left behind due to the unreliability of electricity, delays in accessing materials and difficulty in understanding certain concepts that required verbal explanations. Since there is a need to promote digital learning, compounded by school closures, policies that support digital learning should include ways of addressing bottlenecks to online learning. A randomised trial in Botswana, showed that direct phone calls and SMS text messages interventions were possible low-technology interventions that would complement the traditional education system [30]. These two interventions were cost-effective to include low-earning households, were easy to scale up and highly engaged parents in their children’s educational activities. Future studies should explore how digital training can be innovatively embraced among students and trainers in the context of post-COVID-19 schooling.

The unexpectedly prolonged school closures negatively affected educational institutions including adjustment of academic schedules and programs, reduced incomes for schools and teachers, demotivated teachers and led to bankruptcy and sale of schools. This financial and administrative crisis was experienced in several low-income countries and could have been due to the unpreparedness of schools for closure during the emergency. Some schools, particularly privately owned schools could not cope with the economic shock and either became bankrupt and were sold or repurposed for other activities [31–33]. There is a need for sustainable mechanisms to be instituted to protect schools and teachers from these consequences in the future. This could include policies that protect privately-owned institutions through public-private partnerships in the education sector, social insurance to protect school workers against economic hardships, and support for debt recovery for indebted institutions among others [33–35].

Across the four countries in our study and several other sub-Saharan countries including Kenya, Zimbabwe, and South Africa, there was an increase in school dropouts, teenage pregnancies and increased substance and alcohol abuse as effects of school closures [36–38]. School closures ensured that learners were idle and continuously socialized with peers or youthful friends in their communities compounding these consequences [37, 39] which existed even pre-COVID-19 [40]. In Uganda, which had a prolonged school closure, students experienced depression and anxiety, and spent most of their time in less constructive and passive activities including sleeping, watching television, and browsing social media, which impacted their lifestyles and health [39]. The inequities in education also increased, further disadvantaging students from low-income households, public schools, rural areas, and those with special needs. A multi-stakeholder strategic approach, which embraces the role of government, educational institutions, parents or caregivers, and the local communities [41], is key in mitigating these consequences. Long-term measures will be required to support learners who were left behind and the reintegration of those who may have challenges returning to school. The high drop out of students and poor continuity of learning because of COVID-19 school closures set back Africa’s ability to attain SDG 4, whose goal is to ensure inclusive and equitable quality education and promote lifelong learning opportunities for all. There is therefore a need to monitor the longer-term consequences of school closures and their impact on the education sector and the country’s socio-economic development.

Considering the largely negative consequences of school closures experienced due to COVID-19, the intervention should only be considered as a last resort to minimise their potential negative effects. Response measures that encourage learners to stay in school should be prioritised. Even when schools are closed, it should be for the shortest period possible, and they should be prioritised for reopening as early as feasible while providing adequate protection to learners and their teachers.

### Study limitations and strengths

We conducted a qualitative assessment which does not show the magnitude of the impacts experienced within the country’s leading to a loss of variation between countries as there were differences in the duration of school closures. Moreover, due to the nature of our study, we were unable to interrogate the long-term consequences of school closures. Future studies should consider delving into longer-term consequences and quantifying them including the impact on student performance, completion, and mental health and sexual and reproductive health outcomes among others. Our assessment was also more focused on the consequences at the lower education levels and the picture may be different at the tertiary level whose closure was more short-term in most contexts. On the other hand, we obtained information from various stakeholders including learners, teachers, administrators, and policymakers among others which allowed for the triangulation of study findings.

## Conclusions

School closures as undertaken in DRC, Nigeria, Senegal, and Uganda led to adoption of innovative methods of learning, improved parental involvement in children’s education, and increased engagement of students in extracurricular activities. On the other hand, school closures derailed students’ academic progress, led to school dropouts, reduced school revenue, and reduced teacher incomes, especially in private schools. Overall, the bulk of negative consequences outweighed the positive ones with the potential for both short-term and far-reaching long-term consequences. Although important for infectious disease emergency response, in future pandemics, careful consideration of the type and duration of education closure measures and examination of their potential consequences in the short and long term is important before deploying them. Even when deployed, additional measures that are equitable should be instituted to minimise the negative impact of school closures and schools should be prioritized for reopening as soon as feasible.

## Supporting information

S1 File Interview guide: Key informant interview guide. (DOCX)

S1 Checklist Consolidated criteria for reporting qualitative studies (COREQ): 32-item checklist. (DOC)
