# Supplementary material for: Consequences of school closures due to COVID-19 in DRC, Nigeria, Senegal, and Uganda
Source: PLOS Glob Public Health. 2023 Oct 16;3(10):e0002452. doi: 10.1371/journal.pgph.0002452 (PMC10578567; doi:10.1371/journal.pgph.0002452)
Supplement: S1 File — (DOCX) [file pgph.0002452.s001.docx]

**Consequences of school closures due to COVID-19 in DRC, Nigeria, Senegal, and Uganda**

## Key informant interview guide

1. Identifiers (Institution, Office/Position, Duration in the position, Gender, Profession)
2. How was school and educational institutions closure implemented? Who implemented it?
3. Who was targeted by the school and educational institutions closure? (what were their characteristics e.g. socio-economic status, health status, gender, type of job etc). How did the school and educational institutions closure impact these characteristics? What were the perceptions and attitudes of those targeted by these measures?
4. What were the norms, culture and characteristics (for example, face to face teaching, congested classrooms, term duration, reading culture, interaction among students and teachers etc.) of the school community? How did school and educational institutions closure impact these?
5. To what extent was the school and educational institutions closure adhered to? What factors contributed to the observed extent of adherence? What challenges were faced in ensuring adherence to school and educational institutions closures?
6. How effective would you say the school and educational institutions closures were in supporting the control of the COVID-19 pandemic in the country?
7. What were the positive effects resulting from the school and educational institutions closure at the school, community and population levels? (probe: better hygiene measures, adoption of technology in learning, more time for parents and children / parental involvement in student’s education, reduction in the spread of illnesses)
8. What were the negative effects of school and educational institutions closure at the school, community and population levels? (probe: inefficiency in learning / lack of learning, high costs for learning from home, increased screen time, boredom among children, substance or alcohol abuse among students, teenage pregnancies, school dropouts. poor welfare or standards of living for teachers, teachers leaving profession)
9. What strategies did government and other stakeholders use to minimize the negative effects of the school and educational institutions closure at the school, community and population levels? To whom were the measures targeted and how were they implemented? To what extent did the implemented strategies reduce the negative effects?
10. What mechanisms did individuals themselves adopt to cope with the negative effects of school and educational institutions closure at the school, community and population level?
11. How did the implementation of school and educational institutions closure impact disadvantaged groups at the school, community or population level? (Negatively or positively)

Which groups were impacted? (How about: socially vulnerable communities including those living in slums, the rural poor, refugees, and persons with disabilities?)

1. Considering the period after the school and educational institutions closure was partially or wholly lifted, what other positive and negative effects did it have at the school, community and population level?
2. What would you recommend regarding the implementation of school and educational institutions closure in the control of COVID-19 and pandemics?
